# A Longitudinal Investigation of the Causal Relationship Between Wellbeing and Perceived Discrimination Among Migrant Children in China: The Mediating Role of Self-Esteem and the Moderating Role of School Type

**DOI:** 10.3389/fpsyt.2022.899888

**Published:** 2022-07-08

**Authors:** Qing Wang, Jie Yu, Yuanmeng Tang, Jing Luo, Baoguo Shi

**Affiliations:** Beijing Key Laboratory of Learning and Cognition, School of Psychology, Capital Normal University, Beijing, China

**Keywords:** wellbeing, perceived discrimination, self-esteem, school type, migrant children

## Abstract

**Background:**

A large rural labor force has been attracted to urban areas with the acceleration of urbanization in China. This significant change in environment for migrant children from rural to urban may lead to psychological problems, such as decreased subjective wellbeing (WB) and increased perceived discrimination (PD). However, previous studies have focused on the influence of PD on WB by using a cross-sectional design, ignoring the causality and intrinsic mechanisms between WB and PD. The current study investigates the causal association and internal relations between migrant children’s PD and WB.

**Methods:**

A total of 466 (222 females, 47.64%) migrant children (*M*_*age*_ = 11.78, *SD* = 1.80) were recruited from Beijing in China. The participants filled in the questionnaire twice, with an interval of 1 year, including a basic information questionnaire, wellbeing index scale, perceived discrimination questionnaire, and self-esteem scale.

**Results:**

Overall, cross-lagged regression analysis revealed that WB (T1) had a predictive effect on PD (T2) but that PD (T1) had no predictive effect on WB (T2). Mediation results indicated that self-esteem (SE) (T1) mediated the relation between WB (T1) and PD (T2). Moderated mediation results further proved that the link between WB (T1) on SE (T1) and the indirect effect between WB (T1) and PD (T2) were more robust for migrant children who attended public school than those in the migrant children’s school.

**Conclusion:**

These findings implied that a decrease in WB may increase the perception of subjective discrimination and that SE could be an intrinsic factor between migrant children’s WB and PD, especially in public schools. Therefore, educators and parents should also pay attention to mental health problems to improve the wellbeing and self-esteem of migrant children.

## Introduction

With the acceleration of urbanization and the increasing demand for quality of life in China, a large rural labor force has been pouring into cities and seeking jobs there. This phenomenon has led to another social group—migrant children. Migrant children are children who have left their rural hometowns to live with their families in the city for above 6 months during the compulsory education stage but who have not obtained local household registration in the city (1). Remarkably, the number of migrant children is still increasing. According to the 2020 census, statistics showed that there were 130 million migrant children in China, accounting for 40% of the total population of children in China (2). The change in circumstances, the increasing economic pressure, and the lack of education guarantees in cities may place migrant children in disadvantaged situations, thus resulting in social, emotional, and behavioral problems (3, 4). Based on previous studies, there are two important indicators for measuring the adaptability of migrant children to a new environment: perceived discrimination (PD) and wellbeing (WB) (4). WB is individuals’ overall evaluation of their quality of life, which can serve as an essential indicator of the level of mental health (5, 6). PD, in relation to objective discrimination, is individuals’ subjective sense of being treated differently or unfairly due to the group to which they belong (7). Previous cross-sectional studies have indicated a close relationship between PD and WB (4). Little longitudinal research has explored the causal order and underlying influencing mechanism between WB and PD. Given that integrating migrant children into their cities is important since many factors depend upon it, a more comprehensive understanding of WB and PD is beneficial and necessary in modern society. Thus, the present study utilized a longitudinal sample of migrant children to explore (a) the causal association between migrant children’s WB and PD, (b) whether migrant children’s WB and PD could be mediated by self-esteem (SE), and (c) whether the pathways between WB and PD are moderated by school type (ST).

### The Causal Relationship Between Wellbeing and Perceived Discrimination

Researchers have increasingly explored the correlation between WB and PD from the perspective of different groups. Although numerous studies have shown that WB is closely related to PD, the causal relation between these two variables remains unclear (8, 9). On the one hand, most studies have regarded perceived discrimination as a predictor of wellbeing, including direct roles and indirect effects (4, 10, 11). A meta-analytic study comprehensively explored 110 PD and mental health studies and found that one’s perception of discrimination could significantly negatively predict WB (9). Recent studies also found similar results for migrant children (8). To our knowledge, PD is a low-status group’s social stress based on group membership (e.g., gender and socioeconomic status), separating them from the dominant group (11); thus, migrant children with higher PD are likely to have negative emotions. Such negative emotions may further lead to the loss of migrant children’s WB. However, on the other hand, some researchers maintain that WB is the antecedent of PD, with PD being more susceptible to one’s emotions (12, 13). Based on the broaden-and-build theory, positive emotions allow individuals to build psychological, intellectual, and social resources, whereas negative emotions lead to narrow self-perception and poor interpersonal relationships (14, 15). Wellbeing is an individual’s feelings of positive emotional experience (16). Therefore, migrant children with a low level of WB are more likely to have low self-evaluation, be unable to actively adapt to environmental changes, and experience poor interpersonal relationships (17, 18), which may lead to exaggerated or increased PD. Moreover, mental development in childhood is not mature or stable, and the perception of surrounding social activities is more susceptible to the influence of negative emotions (19). Such negative emotions may further lead to an increase in subjective discrimination perceptions.

Overall, there are different theoretical explanations for the bidirectional relationship between WB and PD. However, previous studies primarily used the cross-sectional method, making the causal relations unclear. Therefore, this study verifies the causal association between WB and PD based on longitudinal data. Based on the evidence reviewed above, we hypothesize that migrant children’s WB and PD mutually affect each other (hypothesis 1).

### The Mediating Role of Self-Esteem

The associations among wellbeing, self-esteem, and discrimination has been widely investigated (10, 20). However, few studies have focused on the role of SE in the longitudinal relationship between PD and WB. Self-esteem (SE), formed by evaluating self-characteristics, is the feeling of self-worth and self-respect (21, 22). According to the identity theory of SE, an individual with a high level of SE can mobilize individual resources and buffer negative emotional experiences. Otherwise, it will aggravate negative emotions (23). Individuals may internalize prejudice caused by discrimination as a part of self-verification, which will lead to more negative views of themselves, thus leading to the loss of WB (24). An empirical study showed that PD hurts SE (7). For example, PD reduces the SE of people with physical disabilities and migrant children (20, 25). In addition, abundant evidence shows that SE can significantly predict WB (20, 26). Hence, PD may influence the WB of migrant children through SE.

Conversely, the WB of migrant children may also influence their PD through self-esteem. As the broaden-and-build theory suggests, positive emotion can help individuals build more psychological skills and resources by enhancing self-efficacy and SE (15, 17); individual WB is associated with positive emotion and involves the overall assessment of individual life (27). Thus, it may also affect the SE covering overall judgment about oneself or social group. For example, a meta-analytic study showed that positive affect influences multiple life domains, including social relationships and positive perceptions of self and others (e.g., SE) (28). In addition, SE could further affect migrant children’s PD. Many empirical studies have found that high SE is related to low PD (10, 29, 30). Moreover, high SE allows people to maintain self-worth and rate themselves more positively (31–33), so SE may be a protective factor against PD (34, 35). Therefore, the wellbeing of migrant children may influence their PD through SE.

Taken together, SE may have a mediating effect on the reciprocal association between WB and PD. Given previous theories and empirical literature, we hypothesize that SE mediates the mutual association between WB and PD among migrant children (hypothesis 2).

### Moderating Role of School Type

School life takes up most of the migrant children’s time, so it is essential to examine the moderation of the relationship between WB and PD. In China, migrant children have access to public schools and migrant children’s schools to receive an education (36, 37). Public schools are organized and funded by the government and have strict requirements for local household registration, so they are dominated by city children (38). Migrant children’s schools were established and funded by the government or individuals to provide education for the children of migrant workers. Compared to migrant children in public schools, migrant children in migrant children’s schools experience greater deficiencies in education resources, education standards, and teacher resources (39).

Although some empirical studies have examined the difference in the SE, PD, and life satisfaction between the children of migrant workers in public schools and those in public schools, the role of school type (ST) in the longitudinal relationship between WB and PD is unclear (40). Notably, migrant children in public schools have a strong sense of urban belonging and are likely to enter into identity crises and social exclusion when integrating into schools (41, 42). Thus, once they feel that city children reject their membership (i.e., belong to the city), their subjective perceptions of discrimination may increase along with negative emotions. In addition, ecological systems theory suggests that individuals experience stress and negative emotions when they perceive themselves in a disadvantageous situation and lack sufficient social support (43). Moreover, migrant children in public schools are prone to have negative emotions related to inferiority and loneliness (44, 45). These adverse emotions also affect individuals’ perceptions of the friendliness of the surrounding environment, such as increased PD (46, 47). As a previous study showed, migrant adolescents’ PD in public schools has a stronger connection with WB than in those in migrant children’s schools (4). Therefore, the link between migrant children’s WB and PD was significantly stronger in public schools than in migrant children’s schools. Given these findings, we hypothesize that ST moderates the mutual association between WB and PD among migrant children (hypothesis 3).

### The Present Study

**FIGURE 1:**
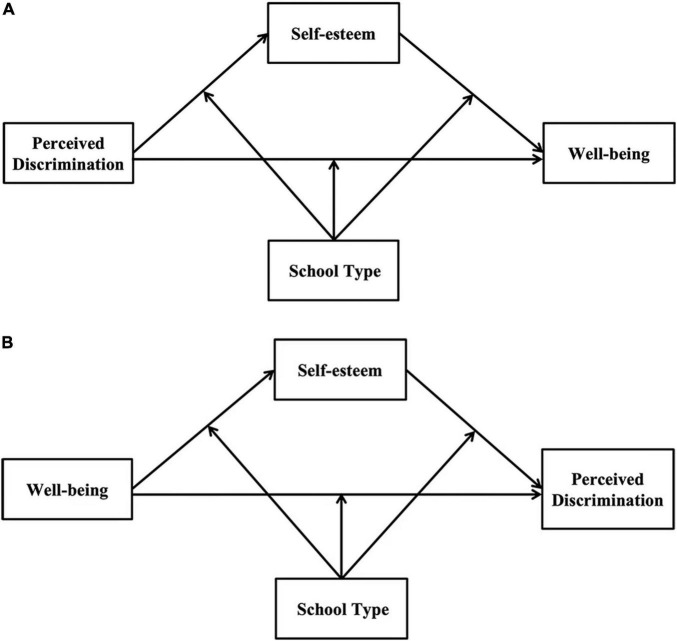
The proposed moderated mediation model. **(A)** The moderated mediation model of perceived discrimination to well-being; **(B)** the moderated mediation model of well-being to perceived discrimination.

Overall, this study explored three key topics. First, the study explored the causal association between WB and PD. Second, the study tested the mediating role of SE on the link between WB and PD. Last, the study tested the moderating role of ST on the link between PD and WB. Figures 1A,B illustrates the research model.

## Materials and Methods

### Participants

The data were acquired through two measurement waves, with an interval time of 1 year. First, a public elementary and middle school and a migrant children’s school were selected by simple random sampling in Beijing, China. Next, 486 migrant children completed the surveys in two waves, and valid data were collected from 466 participants (222 girls, 47.64%) after eliminating incomplete responses and extreme values at T1 and T2. Among them, 224 migrant children (48.07%) were educated at migrant children’s schools. In the first wave, 273 migrant children were pupils in the third (69 migrant children, 14.81%), fourth (83 migrant children, 17.81%) or fifth grade (121 migrant children, 25.97%), and the rest of the migrant children were middle school students in the first (112 migrant children, 24.03%) or second grade (81 migrant children, 17.38%). The average age of the migrant children was 11.78 years (*SD* = 0.91), ranging from 8 to 16.

### Measures

#### Wellbeing Index Scale

Migrant children’s WB was measured by the wellbeing index scale (48), consisting of 9 items. The 9 items were divided into the overall affective index and life satisfaction. All the nine items were rated on a 7- point Likert scale (1 = disagree entirely, and 7 = agree entirely). The total score was the average score of part I (8 items) added to the score of Part II (1 item) according to a 1:1 ratio. On this scale, higher scores suggest a higher level of WB. This scale was widely applicable in various groups (49). In the present study, the scale shows good reliability (Cronbach’s α is 0.79∼0.88).

#### Perceived Discrimination Questionnaire

Migrant children’s PD was measured by a perceived discrimination questionnaire (50), consisting of 21 items. The 21 items covered speech discrimination, bodily assault, and avoidance. Each item was rated on a 5-point Likert scale (1 = disagree strongly, and 5 = agree strongly). On this questionnaire, higher total scores suggest a higher level of PD. This scale is widely used to investigate the discrimination perception of migrant children in China (8). In the present study, the questionnaire shows good reliability (Cronbach’s α is 0.85∼0.80).

#### The Rosenberg Self-Esteem Scale

Migrant children’s SE was assessed by the Rosenberg Self-Esteem Scale (21) consisting of 10 items. The participants rated each item on a 4-point Likert scale (1 = not very true of me, and 4 = very true of me). On this scale, higher total scores suggest a higher level of SE. The scale was proven to be suitable for children aged 7–12 years and migrant children in China (51, 52). In the present study, the scale shows good reliability (Cronbach’s α is 0.80).

#### The Socioeconomic Status of Families

The socioeconomic status (SES) of families was measured by parents’ occupation and education level (53). The parents’ occupations were separately coded into five grades according to the standard of occupational classification (1 indicating “temporary workers, the unemployed, unskilled workers and farmers” and 5 indicating “senior manager, senior technicians and the professional supervisor”) (54). The parents’ education levels were converted into six grades (1 indicating “uneducated” and 6 indicating “graduate degree”). The SES of the family equals the sum of the parents’ occupation grade and education grade, and the score ranges from 4 to 22, with a higher score representing a higher families SES.

### Procedure

This study followed the principles of the Institutional Review Board of the author’s university and the participating school. First, we sought consent from the participants and their parents. Next, we informed the participants that they could withdraw at any time, and the survey was anonymous to ensure the truthfulness of their responses before completing the questionnaire. Third, the participants finished self-report scales. We provided specific guidance for third-grade pupils to fill out the scales. It took 10 min to complete the survey. Finally, the information on schools was obtained from the official website of the Beijing Municipal Education Commission, and the schools were classified as public schools or migrant children’s schools.

### Statistical Analyses

We conducted data analysis with SPSS 22.0 and Mplus 8.0. All models were adjusted for gender, age, and family SES. The fit indexes were used to assess the overall fit of the models, and the models were acceptable if the normed chi-square model (χ^2^/df) < 5, the root mean square error of approximation (RMSEA) < 0.08, and the comparative fit index (CFI) > 0.95 (55).

First, all data were analyzed by Harman’s single-factor to test for common method bias. Second the multiple imputation method (MIm) was conducted to estimate missing data to accurately obtain parameter estimation results (56, 57). Third, descriptive statistics and partial correlations of all variables were used to perform the preliminary analyses. Fourth, the cross-lagged model was tested to verify the causal associations between WB and PD. Fifth, the mediation model was examined by a four-step procedure (58, 59), which required that (a) the migrant children’s WB was significantly associated with PD, (b) the migrant children’s WB was significantly associated with SE, (c) the migrant children’s SE was significantly associated with PD, and (d) the indirect path between WB and PD *via* SE was significant. In addition, the indirect effect needs to be justified by 5,000 bootstraps to obtain the 95% confidence interval (CI) of parameter estimation and is statistically significant if the confidence interval does not contain 0 (60). Finally, the moderated mediation was examined to estimate the moderating effect of ST on the mediation model of the migrant children’s WB and PD. It required the interaction effect to be significant, and the 95% CI of the interaction effect did not contain 0. Moreover, the effect of variables (WB or SE) no interaction with ST on PD is fixed to be independent of W and any other variable in the model (61). In addition, we further analyze the conditional results and plot the slope diagram to present the difference between public schools and migrant children’s schools in the indirect effect and all paths between WB and PD.

## Results

### Common Method Biases

Harman’s single-factor method was a reliable method to examine whether common method biases exist in this study. The results showed that 10 eigenvalues greater than one were extracted from the unrotated exploratory factor analysis. Moreover, 20.69% of the variation was explained in the first eigenvalues, which do not reach a critical value (40%). Thus, this study did not have severe common method bias (62).

### Missing Data

The raw data were relatively complete, and they were missing at random. Therefore, we used the multiple imputation method (MIm). The number of imputations on the missing data was 50, given that previous studies have pointed out that the more interpolations there are, the more accurate the estimation and that 20 interpolations are acceptable (63).

### Preliminary Analyses

Table 1 reported the mean, *SD* and partial correlation coefficients among variables. The partial correlational coefficients excluded the influence of gender, age and family SES. The results were consistent with our expectations. The repeated measures of WB and PD were positively correlated over time among migrant children (*r* = 0.30, *p* < 0.001) (*r* = 0.54, *p* < 0.001). In addition, the migrant children with high levels of WB at T1 were likely to generate low levels of PD at T1 (*r* = –0.30, *p* < 0.001) and T2 (*r* = –0.36, *p* < 0.001). Similarly, the migrant children with high levels of WB at T2 were likely to generate low levels of PD at T1 (*r* = –0.17, *p* < 0.001) and T2 (*r* = –0.26, *p* < 0.001). The migrant children with high SE at T1 were likely to generate high WB and low PD at T1 (*r* = 0.30, *p* < 0.001) (*r* = –0.42, *p* < 0.001) and T2 (*r* = 0.19, *p* < 0.001) (*r* = –0.32, *p* < 0.001).

**TABLE 1:** Descriptive statistics and partial correlations between variables (*N* = 466).

	*M (SD)*	1	2	3	4	5
1. T1 WB	9.89 (3.27)	1				
2. T2 WB	9.81 (3.41)	0.30***	1			
3. T1 PD	48.06 (11.26)	−0.30***	−0.17***	1		
4. T2 PD	45.84 (12.44)	−0.36***	−0.26***	0.54***	1	
5. T1 SE	28.45(3.78)	0.30***	0.19***	−0.42***	−0.32***	1

PD, Perceived discrimination; WB, Wellbeing; SE, Self-esteem; T1, Time 1; T2, Time 2; ***p < 0.001.

### The Causal Relationship of Wellbeing and Perceived Discrimination

**FIGURE 2:**
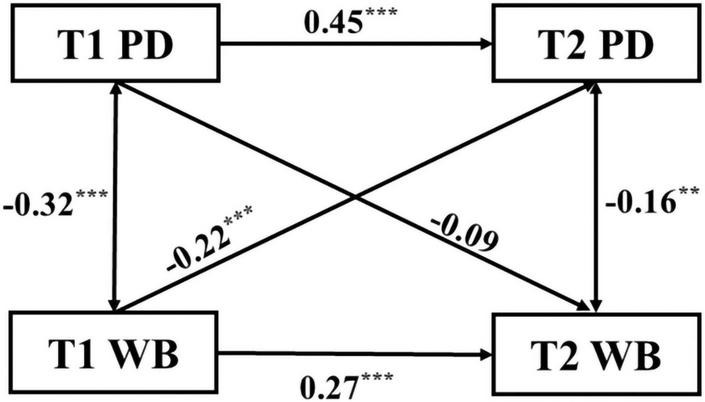
Cross-lagged model of perceived discrimination and wellbeing. PD, Perceived discrimination; WB, Wellbeing; T1, Time 1; T2, Time 2; **p < 0.01; ***p < 0.001.

Hypothesis 1 assumed that WB and PD mutually affect each other. Model fit indexes for the cross-lagged model were saturated (χ^2^ = 0.000, RMSEA = 0.000, CFI = 1.000), indicating that the model was acceptable. The cross-lagged model between the migrant children’s WB and PD was presented in Figure 2. First, path analysis indicated that the migrant children’s WB at T1 significantly and positively predicted WB at T2 (β = 0.27, *p* < 0.001). The migrant children’s PD at T1 significantly and positively predicted PD at T2 (β = 0.45, *p* < 0.001). Second, the migrant children’s WB was significantly negatively correlated with PD at the two time points (β = –0.32, *p* < 0.001) (β = –0.16, *p* = 0.002). Finally, path analysis indicated that the migrant children’s WB at T1 significantly and negatively predicted later PD (T2) (β = –0.22, *p* < 0.001). However, the migrant children’s PD at T1 did not predict later WB (T2) (β = –0.09, *p* = 0.074). These results indicated that the migrant children’s WB was a stable antecedent variable of PD. Thus, Hypothesis 1 was partially rejected.

### Test of the Mediating Effect

Hypothesis 2 assumed that SE (T1) mediates the relationship between WB (T1) and PD (T2). Model fit indexes for the cross-lagged model were saturated (χ^2^/*df* 0.000, RMSEA = 0.000, CFI = 1.000), indicating that the model was acceptable. The mediation results of SE between the migrant children’s WB and PD were presented in Table 2. First, Model 1 showed that the migrant children’s WB (T1) was significantly negatively related to their PD (T2) (β = –0.35, *p* < 0.001). Second, Model 2 showed migrant children’s WB (T1) was significantly positively related to SE (T1) (β = 0.30, *p* < 0.001), and Model 3 showed that SE (T1) was negatively related to PD (T2) (β = –0.22, *p* < 0.001). Last, the indirect effect of WB on PD (T2) corrected by 5000 bootstrap was significant (*indirect effect* = –0.07, *SE* = 0.02, 95% *bias-corrected CI* = [–0.10, –0.04]). Therefore, the mediation effects of SE between WB and PT were fully proved according to the four criteria. Therefore, the results partly supported Hypothesis 2.

**TABLE 2:** Testing the mediating effect of self-esteem (T1) on perceived discrimination (T2).

	Model 1 (T2 PD)				Model 2 (T1 SE)				Model 3 (T2 PD)			
			
	β	*t*	*LLCI*	*ULCI*	β	*t*	*LLCI*	*ULCI*	β	*t*	*LLCI*	*ULCI*
T1 WB	–0.35	−8.11***	–0.42	–0.28	0.30	6.78***	0.23	0.37	–0.28	−6.25**	–0.36	–0.21
T1 SE									–0.22	−4.89***	–0.30	–0.15
*R* ^2^	0.21				0.13				0.26			
*F*	5.91***				4.07***				6.91***			

All models controlled for gender, age, SES; PD, Perceived discrimination; WB, Wellbeing; SE, Self-esteem; T1, Time 1; T2, Time 2; **p < 0.01; ***p < 0.001.

### Test of Moderated Mediation

**FIGURE 3:**
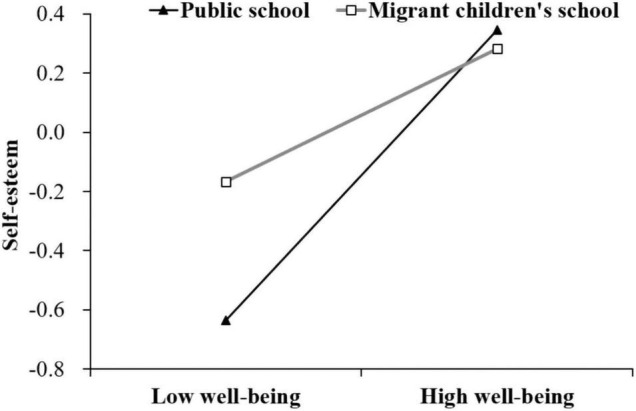
School type moderates the relationship between self-esteem (T1) and wellbeing (T1) for public school and migrant school children.

Hypothesis 3 assumed that ST moderates the relationships between WB and PD (Figure 1). The model fit indexes of the moderated mediation model were poor (χ^2^/*df* = 56.54 > 5, RMSEA = 0.35 > 0.08, CFI = 0.90 < 0.95). As shown in Table 3, this effect between the migrant children’s WB (T1) and PD (T2) was not moderated by ST (β = 0.16, *p* = 0.32), and this effect between SE (T1) and PD (T2) was not moderated by ST (β = 0.42, *p* = 0.16). Last, the effect between the migrant children’s WB (T1) and SE (T1) was significantly moderated by ST (β = –0.49, *p* = 0.006). Moreover, the migrant children’s SE (T1) was significantly negatively related to their PD (T2) (β = 0.40, *p* < 0.001). For clarity, we plotted WB (T1) on SE (T1) separately at the public school and migrant children’s school (see Figure 3). The simple slope analyses found that the effect between WB and SE (T1) was stronger for the migrant children in public school (β_*simple*_ = 0.44, *p* < 0.001) than for those in the migrant children’s school (β_*simple*_ = 0.26, *p* = 0.002). The mediating effect of WB (T1) on PD (T2) through SE (T1) was moderated by ST. Specifically, the indirect effect was stronger for the migrant children in public school (β = –0.14, *SE* = 0.04, 95% *CI* = [–0.20, –0.08]) than for those in the migrant children’s school (β = –0.05, *SE* = 0.02, 95% *CI* = [–0.09, –0.01]). Thus, these results partly supported Hypothesis 3.

**TABLE 3:** Testing the moderated mediation effect of wellbeing (T1) on perceived discrimination (T2).

Predictors	T1 SE				T2 PD			
		
	β	*t*	*LLCI*	*ULCI*	β	*t*	*LLCI*	*ULCI*
T1 WB	0.76	4.82***	0.50	1.02	–0.27	–1.89	–0.53	–0.03
ST	0.53	3.34**	0.27	0.79	–0.20	–0.68	–0.65	0.26
T1 WB × ST	–0.49	–2.73**	–0.78	–0.19	0.16	0.88	–0.11	0.43
T1 SE					–0.40	–3.76***	–0.57	–0.22
T1 SE × ST					0.42	1.42	–0.07	0.91
*R* ^2^	0.15				0.43			
*F*	4.68***				5.68***			
								

**Indirect effect**	**β**		***Boot SE***		***t***		***BOOT LLCI***	***BOOT ULCI***

Public school	–0.14		0.04		–3.98***		–0.20	–0.08
Migrant children’ school	–0.05		0.02		–2.21*		–0.09	–0.01

All models controlled for gender, age, SES; PD, Perceived discrimination; WB, Wellbeing; ST, School type; SE, Self-esteem; T1, Time 1; T2, Time 2; *p < 0.05; **p < 0.01; ***p < 0.001.

## Discussion

Most studies of PD and WB have suggested only a correlational relationship between the variables; however, few studies have made strong causal inferences and further explored the underlying influencing mechanism between PD and WB. To fill this gap, a cross-lagged model was used to verify the causal association between PD and WB; a moderated mediation model was used to examine whether the causal association could be mediated by SE and moderated by ST in a two-wave survey. The results showed that the migrant children’s WB (T1) strongly affected their PD (T2). Moreover, SE (T1) mediated the relationship between the migrant children’s WB (T1) and PD (T2), and the link between WB (T1) and SE (T1) are robust in public school. We discussed these finding separately in the following parts.

### Wellbeing as a Predictor of Perceived Discrimination

The study proved that migrant children’s WB stably and negatively predicts their PD *via* the two-wave tests. Nevertheless, the results are unable to support the opposite direction. Thus, the results partially rejected the hypotheses that migrant children’s WB and PD are bidirectional and supported that migrant children’s WB was a stable antecedent variable of PD. This finding is interesting because the migrant children’s WB predicted PD 1 year later. Previous studies have shown that one’s perception of discrimination can lead to the loss of psychological WB (4, 9, 10, 64). In addition, a meta-analytic review found that PD has a causal effect on WB (13). This result is somewhat counterintuitive. However, it is remarkable that most previous studies on individual WB and PD were cross-sectional studies that provided only correlational data, thus limiting the ability to make causal inferences. Moreover, compared to objective encounters with discrimination, PD is the subjective perception that one faces discrimination, and it is more susceptible to one’s emotion (12, 13). On the one hand, longitudinal studies showed that WB precedes a host of other desirable outcomes, including good health, prosocial behavior, academic satisfaction, and school achievement (65–67). Moreover, WB has been shown to improve the quality of interpersonal relationships and intimacy and increase prosocial behavior (14, 68, 69). In other words, compared with children with low WB, migrant children with high WB are better at socializing and establishing friendly relations with others and experience less discrimination. On the other, adverse emotions limited one’s attention to support specific action tendencies (15, 70). That is, the reduced WB of migrant children due to the sudden changes in their living environment is likely to lead to negative impressions of the surrounding environment and group and exaggerated perceptions of discrimination. Therefore, migrant children’s WB could be a stable predictor of PD. The finding illustrated the causal association between migrant children’s PD on WB which go beyond the cross-sectional studies. Although this finding is somewhat counterintuitive, it emphasizes the importance of the influence of individuals’ positive emotions on their perceptions of themselves and the external world.

### The Mediating Role of Self-Esteem

The current funding proved that SE mediates the link between migrant children’s WB and PD. These findings explain how the reduction of migrant children’s WB can lead to an increase in PD. That is, migrant children’s WB is positively associated with SE, and SE is negatively associated with migrant children’s PD. As we all know, SE is a leading factor affecting happiness based on most cross-sectional studies, which can only prove that WB and SE are related (71–73). In addition, a longitudinal study also revealed that SE affects happiness (74). However, the main weakness of previous studies is ignoring the potential impact of individuals’ WB on all aspects of psychology and behavior (27). For example, individuals with a high sense of WB have higher self-evaluations, are less self-critical, and have higher SE (75, 76). In contrast, individuals with more negative affect appear to have lower SE (77). Moreover, children’s subjective judgments are more easily affected by their emotions (78). Thus, migrant children’s WB may affect their SE. Another possible explanation for this is that most migrant children are forced to follow their parents to live in cities due to economic pressure, so the loss of WB may be inseparable from the economic pressure on their families (79). As previous research suggests, economic WB is one of the essential factors in developing and cultivating SE among young adults (80). Migrant children’s family economic status is generally low compared with that of urban children (81). Therefore, the loss of WB related to economic pressure may decrease migrant children’s SE.

In addition, migrant children’s SE also affects their perceptions of discrimination. On the one hand, migrant children with high SE can identify self-worth better than migrant children with low SE and thus have weaker subjective feelings of external objective discrimination (31–33). On the other hand, SE is associated with peer relationships and peer acceptance (82, 83). This also leads to migrant children with high SE experiencing less discrimination. Therefore, a low level of SE can explain the reason for why migrant children with a low level of WB are likely to have strong perceptions of discrimination. More importantly, there could be a vicious circle between the low WB of migrant children and PD. Consequently, it would be helpful for educators and parents to apply this theory to improve migrant children’s WB and SE and reduce PD.

### The Moderating Role of School Type

The current study revealed that ST moderates the link between migrant children’s WB and SE and the indirect path between WB and PD. Specifically, the effects were stronger in public schools, which suggests that the migrant children’ SE and PD was more vulnerable to WB when they are educated in public schools. As previous research proved, the association between migrant adolescents’ adverse events and SE is more salient in public schools than migrant children school (4). As mentioned before, migrant children educated in public schools have a more robust demand for self-identity transformation (84). They are more likely to form upward comparisons, resulting in a loss of SE and psychological gaps, leading to a strong link between WB and SE (85). Taken together, the results reveal that ST plays a pivotal role on affecting the connection between migrant children’s WB and SE. It also shed light on the targeted prevention and intervention process should be applied for migrant children in different environments, especially those in disadvantaged situations.

Contrary to our expectation, ST does not moderate the link between migrant children’s WB and PD. Moreover, it does not moderate the link between migrant children’s SE and PD. This may be because both migrant children’s WB and SE have robust effects on PD whatever kind of school they attend. As we mentioned in the cross-lagged regression, WB can significantly negatively predict PD. In addition, previous studies have also indicated that WB and SE are consistently negatively related to PD (25, 86). Therefore, migrant children’s WB and SE significantly predict PD regardless of whether they are in public schools or migrant children’s schools.

### Limitations and Implications

There are several limitations of this research. First, this study assessed children in only two waves, which may have influenced a comprehensive understanding of the link between migrant children’s WB and PD. Additionally, we measured the migrant children’s SE only in the first wave, so it is difficult to draw any causal conclusion between WB and SE. Most likely, there is a bidirectional association between them. Second, the data in this study were based on self-reports, so researchers may be able to collect data from multiple informants, such as the participants’ parents or teachers, in the future to confirm the findings. Third, the study examined Chinese migrant children. However, it focused on exploring the influence of WB on PD. It can also limit the generalizability of the research results. Therefore, future studies can focus more on collecting data from diverse groups.

Despite the limitations mentioned above, the present study makes several key contributions. First, this study proves that a decrease in individual WB increases the perception of objective discrimination by longitudinal survey and emphasizes the significance of positive emotions. Second, the results provide estimable data and theoretical reference for targeted interventions to improve personal WB and decrease PD. Third, the present study underscore the important role of SE between WB and PD, especially in public schools. Therefore, it is important for educators to guide migrant children to gradually integrate into cities to promote their SE and reduce their subjective perception of discrimination.

## Conclusion

Overall, this work contributes to existing knowledge by providing the causal relationship as well as the moderated mediation model of PD and WB, which provides a comprehensive understanding of how migrant children’s WB affects their PD. The results reveal that the migrant children’s WB is a stable antecedent of PD. In addition, migrant children’s SE plays a mediating role in the link between WB and PD, especially for migrant children in a public school.

## Data Availability Statement

The raw data supporting the conclusions of this article will be made available by the authors, without undue reservation.

## Ethics Statement

The studies involving human participants were reviewed and approved by the Institutional Review Board of the Capital Normal University. Written informed consent to participate in this study was provided by the participants’ legal guardian/next of kin.

## Author Contributions

QW analyzed the data, wrote the first draft of the manuscript, and revised the manuscript. JY and YT wrote the first draft of the manuscript and organized the database. JL designed the study and revised the manuscript. BS designed the study, analyzed data, and revised the manuscript. All authors contributed to manuscript revision, read, and approved the submitted version.

## Conflict of Interest

The authors declare that the research was conducted in the absence of any commercial or financial relationships that could be construed as a potential conflict of interest.

## Publisher’s Note

All claims expressed in this article are solely those of the authors and do not necessarily represent those of their affiliated organizations, or those of the publisher, the editors and the reviewers. Any product that may be evaluated in this article, or claim that may be made by its manufacturer, is not guaranteed or endorsed by the publisher.
